# Cloning and expression analysis of two distinct HIF-alpha isoforms – gcHIF-1alpha and gcHIF-4alpha – from the hypoxia-tolerant grass carp, *Ctenopharyngodon idellus*

**DOI:** 10.1186/1471-2199-7-15

**Published:** 2006-04-20

**Authors:** Sheran HW Law, Rudolf SS Wu, Patrick KS Ng, Richard MK Yu, Richard YC Kong

**Affiliations:** 1 Department of Biology and Chemistry, City University of Hong Kong, Kowloon Tong, Hong Kong Special Administrative Region, People's Republic of China; 2 Centre for Coastal Pollution and Conservation, City University of Hong Kong, Kowloon Tong, Hong Kong Special Administrative Region, People's Republic of China

## Abstract

**Background:**

Hypoxia-inducible factors (HIFs) are involved in adaptive and survival responses to hypoxic stress in mammals. In fish, very little is known about the functions of HIFs.

**Results:**

We have cloned and characterized two distinct HIF-alpha cDNAs – gcHIF-1alpha and gcHIF-4alpha – from the hypoxia-tolerant grass carp. The deduced gcHIF-1alpha protein is highly similar to the HIF-1alphas (57–68%) from various vertebrate species, while gcHIF-4alpha is a novel isoform, and shows an equivalent degree of amino acid identity (41–47%) to the HIF-1alpha, HIF-2alpha and HIF-3alpha proteins so far described. Parsimony analysis indicated that gcHIF-4alpha is most closely related to the HIF-3alpha proteins. Northern blot analysis showed that mRNA levels of gcHIF-1alpha and gcHIF-4alpha differ substantially under normoxic and hypoxic conditions, while Western blot studies demonstrated that the endogenous protein levels for both gcHIF-1alpha and gcHIF-4alpha are similarly responsive to hypoxia. Our findings suggest that both gcHIF-1alpha and gcHIF-4alpha are differentially regulated at the transcriptional and translational levels. HRE-luciferase reporter assays show that both proteins function as transcription activators and play distinct roles in modulating the hypoxic response in grass carp.

**Conclusion:**

There are at least two distinct HIF-alpha isoforms – gcHIF-1alpha and gcHIF-4alpha – in the hypoxia-tolerant grass carp, which are differentially expressed and regulated in different fish organs in response to hypoxic stress. Overall, the results suggest that unique molecular mechanisms operate through these two HIF-alpha isoforms, which underpin the hypoxic response in the hypoxia-tolerant grass carp.

## Background

Hypoxia-inducible factors (HIFs) are highly conserved proteins, found in a wide range of animals, that mediate a variety of adaptive cellular and systemic responses to hypoxia by upregulating the expression of more than 60 different genes [1] to assist animals in their adaptation and survival. HIF is a heterodimeric DNA-binding complex consisting of α and β (also known as aryl hydrocarbon receptor nuclear translocator, or ARNT) subunits which are members of the bHLH-PAS (PER-ARNT-SIM) superfamily of proteins [2]. In mammals, three types of HIF-α s (HIF-1α, -2α and -3α) have been reported (for a review see [3]), amongst which the HIF-1α is believed to be the principal regulator of oxygen homeostasis, which heterodimerizes with ARNT and binds to the hypoxia-responsive elements (HREs) of numerous hypoxia-inducible genes to trigger their expression [4]. The increase in HIF-1 activity is primarily due to the hypoxia-induced stabilization and activation of the HIF-1α subunit which is degraded by the ubiquitin-proteasome system under normoxic conditions [5]. This process is activated by the hydroxylation of two conserved proline residues (Pro-402 and Pro-564) within the oxygen-dependent degradation (ODD) domain of HIF-1α by oxygen-dependent prolyl-4 hydroxylases (PHD) [6,7]. Hydroxylation of these proline residues increases the affinity of HIF-1α for the Von Hippel Lindau tumor suppressor (VHL) protein, which is part of an E3 ubiquitin-ligase complex that targets the protein for proteasomal degradation [8]. Moreover, hydroxylation of a conserved asparagine residue (Asn-803) within the HIF-1α C-terminal activation domain by an asparaginyl hydroxylase, known as Factor Inhibiting HIF-1 (FIH), under normoxic conditions inhibits the recruitment of the CBP/p300 coactivator, an important component for HIF-dependent transcriptional activation [9].

Fish are ideal models to study molecular and cellular adaptation to hypoxia because fluctuations in environmental oxygen availability have played an important role in the evolution of these animals [10]. Indeed, compared to mammals, the chronic and protective responses to hypoxia in fish are considerably more diverse [11]. Although HIF-1α and HIF-2α cDNAs have been described in rainbow trout [12] and Atlantic killifish [13], respectively, the presence and function of these molecules in cyprinids, which are generally hypoxia-tolerant, has previously not been studied in detail, although sequences of zebrafish HIFs are available.

In order to obtain broader insights into the evolution and possible functions of the HIF-αs in fish, we have identified and characterized two distinct HIF-α isoforms – gcHIF-1α and gcHIF-4α – from the hypoxia-tolerant grass carp. Here, we present evidence for the differential roles of these two HIF-α proteins in grass carp for adaptation to hypoxia based on their *in vivo *expression and response patterns to short- and long-term hypoxia, and *in vitro *gene transactivation studies. Moreover, parsimony analysis (amongst other findings) support the notion that HIF-4α (so far found only in fish) is possibly an ortholog of the mammalian HIF-3α.

## Results

### Identification and cloning of full-length gcHIF-1α and gcHIF-4α cDNAs

**Figure 1:**
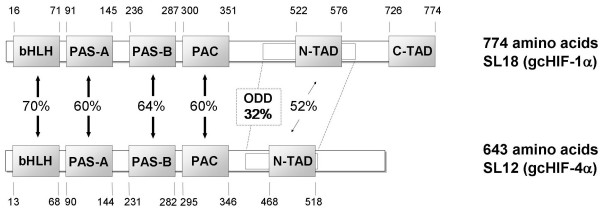
**Domain structures of the inferred gcHIF-1α and gcHIF-4α proteins**. Sequence identities between homologous domains of the two HIF-α isoforms encoded by cDNA clones, SL18 and SL12, are shown. The amino acid positions delineating the different domains are indicated and include: bHLH, basic helix-loop-helix domain; PASA/B, Per-ARNT-Sim A/B domains; PAC, domain C-terminal to PAS motifs; ODD, oxygen dependent degradation domain; N-TAD, N-terminal transactivation domain; and C-TAD, C-terminal transactivation domain.

In an attempt to isolate HIF-α-like cDNA sequences from grass carp, degenerated primers targeting consensus sequences derived from multiple alignment of the bHLH/PAS domains of human [GenBank:AF208487], cow [GenBank:AB018398], rat [GenBank:Y09507] and rainbow trout [GenBank:AF304864] HIF-1α s were used in RT-PCR on total RNA from kidney of grass carp that was exposed to 4 h hypoxia. Two distinct cDNA fragments, c12 (0.45 kb) and c18 (1.8 kb), with partial open reading frames (ORFs) that shared high sequence identity with the HIF-α proteins (bHLH-PAS domains) of various vertebrates species were identified. Using 5' – and 3' -RACE PCR, full-length cDNAs were derived for c12 (2092 bp) and c18 (3848 bp), the authenticity of which were confirmed by full-length RT-PCR on poly (A)^+ ^RNA isolated from grass carp kidney, and were cloned into the pGEM-T plasmid vector to produce the corresponding full-length cDNA clones, SL12 and SL18 (Fig. 1). The clones were completely sequenced on both strands and computer analyses indicated that SL18 contains 5' – and 3' -untranslated (UT) regions of 257 and 1238 bp, respectively, and an ORF that specifies a protein of 774 amino acids with a predicted *M*r of 86 kDa, and is consistent with the detection of a single mRNA transcript of ca. 3.8 kb by Northern blot analysis (data not shown). SL12 contains 5' – and 3' -UT regions of 102 and 29 bp, respectively, and an ORF that encodes for a protein of 643 amino acids with a predicted *M*r of 76 kDa. Northern blot analysis using either the 5' -UT or 3' -end (350 bp) sequences of SL12 as probes indicated that SL12 is expressed in two forms in hypoxic kidney, a major 3.7-kb transcript and a minor 2.1-kb transcript; with the larger transcript showing a 10-fold higher expression level than the former (data not shown).

**Figure 2:**
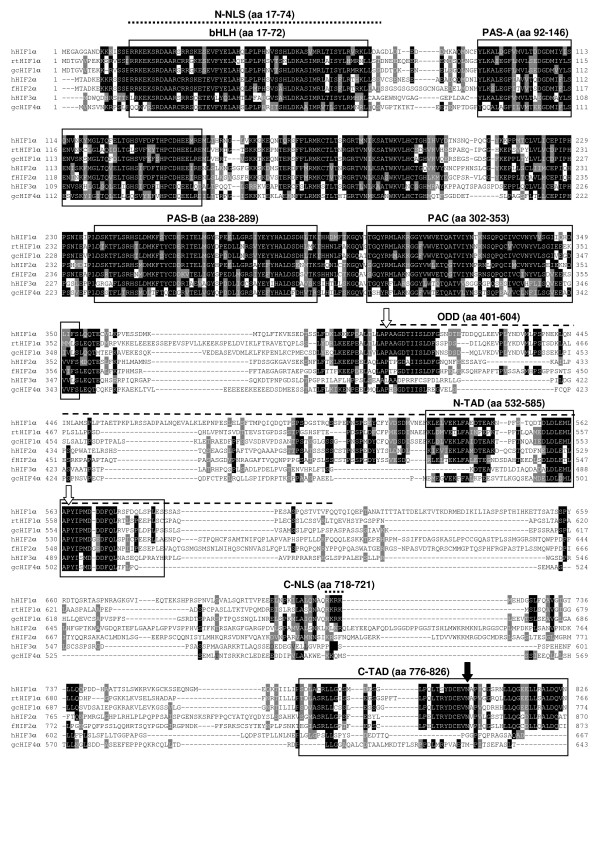
**Multiple alignment of the deduced amino acid sequences of gcHIF-1α and gcHIF-4α with selected human and fish HIF-α homologues**. Amino acids are designated by single-letter codes. From top to bottom: gcHIF1α, grass carp HIF-1α rtHIF1α, rainbow trout HIF-1α; hHIF1α, human HIF-1α; fHIF2α, *Fundulus heteroclitus *HIF-2α; hHIF2α, human HIF-2α; hHIF3α, human HIF-3α; gcHIF4α, grass carp HIF-4α. The number on the left of each row denotes the amino acid position. Identical amino acids shared by most of the HIF-α s are shaded in black while similar amino acids are shaded in grey. Dashes (--) indicate gaps inserted for improved alignment. Domains that are typical characteristic of HIF-α proteins are marked on the alignment according to the amino acid positions in human HIF-1α. The two conserved proline residues within the ODD domain are indicated by open arrows (⇩). The closed arrow () indicates the asparagine residue (Asn-803) in C-TAD which controls HIF-1 binding to CBP/p300.

Database searches using BLASTX showed that the ORF of SL18 shares high amino acid identity with the HIF-1α s of human (64%, [GenBank:AF208487]), cow (65%, [GenBank:AB018398]), rat (64%, [GenBank:Y09507]) and rainbow trout (68%, [GenBank:AF304864]), and moderate (53% with human HIF-2α, [GenBank:BC051338]; 52% with *Fundulus *HIF-2α, [GenBank:AF402782]) to low (45% with human HIF-3α, [GenBank:AB054067]) sequence identity with other HIF-α proteins from various vertebrate species. Moreover, the primary structure of the deduced protein of SL18 contains the characteristic bHLH, PAS-A/B, N-TAD and C-TAD domains (Fig. 1) that are highly similar to homologous domains in the human and rainbow trout HIF-1α proteins (Fig. 2). Taken together, these data indicated that SL18 encodes for the grass carp HIF-1α protein, hereupon designated as gcHIF-1α. In contrast, BLAST analysis of the ORF of SL12 showed that it shares an equivalent degree of sequence identity (41–46%) with the HIF-1α, HIF-2α and HIF-3α proteins from different animal species; which suggests that SL12 does not belong to any of the known HIF-α subtypes documented thus far. Furthermore, although the deduced SL12 protein contains the characteristic bHLH, PAS-A/B, PAC and N-TAD domains, the amino acid sequence at the C-terminal end is highly divergent from those of the HIF-1α, HIF-2α and HIF-3α proteins (Fig. 2). Overall, primary sequence analyses indicated that SL12 highly likely encodes for a novel HIF-α protein, hereupon designated as gcHIF-4α.

### Characteristics of the deduced gcHIF-1α and gcHIF-4α proteins

Sequence alignment of the deduced gcHIF-1α and gcHIF-4α proteins with the rainbow trout HIF-1α (rtHIF-1α), *Fundulus *HIF-2α (fHIF-2α) and human HIF-1α, -2α and -3α proteins indicated extensive sequence similarity in the bHLH, PAS-A/B, PAC and N-TAD domains (Fig. 2). Importantly, several functionally important sequence motifs (that mediate HIF-α subcellular translocation and stability) described in all known members of the HIF-α protein family are also found in the gcHIF-1α and gcHIF-4α deduced proteins. They include (amino acid numbering according to human HIF-1α): the N-terminal nuclear localization signal (N-NLS; residues 17–74) that mediates nucleocytoplasmic trafficking of the HIF-α protein [14,15]; Proline-402 residue, a critical hydroxylation site that mediates HIF-1α degradation under normoxia [7]; Pro-564, Tyr-565 and Ile-566 which are highly conserved in the pVHL recognition sequence PYIXXDDDFXL (residues 564–574) within the N-TAD domain [6] that control ubiquitin/proteasome degradation of HIF-1α under normoxic conditions [16]; and Leu-574, a molecular determinant of Pro-564 hydroxylation that modulates oxygen-dependent proteolysis of HIF-1α [17]. Moreover, gcHIF-1α shares remarkably high sequence similarity with the human HIF-1α and -2α proteins in the C-TAD region, in particular the leucine-rich hydrophobic domain encompassing Asn-803 (an asparagine hydroxylase target site), and Leu-795, Cys-800, Leu-818 and Leu-822 residues which are required for physical interaction of the HIF-α protein with the CBP/p300 transactivators [9] to activate HIF-1 transcriptional activity [18,19]. In addition, Val-802, which correctly positions Asn-803 in HIF-1α for hydroxylation by the asparagine hydroxylase enzyme [20], is also conserved in gcHIF-1α. In contrast, gcHIF-4α shares very little sequence identity in its C-terminal region with the C-TADs of human HIF-1α, -2α or -3α (Fig. 2). Curiously, gcHIF-4α contains a stretch of glutamic acid-rich region (residues 365–397 of gcHIF-4α) that is not found in the HIF-1α, -2α or -3α deduced proteins (Fig. 2).

### Phylogenetic analysis

In order to obtain additional HIF-α homologues from other fish species for phylogenetic analysis, the *Fugu rubripes *(*Takifugu rubripes*) genome [21], medaka (*Oryzias latipes*) genome [22], GenBank/EMBL and zebrafish EST databases [23,24] were queried using TBLASTN. Three distinct HIF-α-like proteins were identified from the *Fugu *genome: one (located in Scaffold2916) shared 71% and 81% sequence identity with the human and rainbow trout HIF-1α, respectively; the second (in Scaffold159) shared 76% and 81% identity with the human and *Fundulus *HIF-2α, respectively; and the third (in Scaffold266) shared 71% identity with gcHIF-4α. Similarly, three distinct HIF-α-like proteins were found in zebrafish: one [GenBank:AAH46875] shared 77% identity with human HIF-1α; the second [GenBank:BX248102] shared 79–80% identity with the mammalian HIF-2α s; and the third [GenBank:CA471737] shared 90% identity with gcHIF-4α. Based on amino acid identity, putative HIF-1α homologues from scaleless carp (*Gymnocypris przewalskii*; [GenBank:AY745735] and grouper (*Epinephelus coioides*; [GenBank:AY735010]; and putative HIF-4α homologues from freshwater pufferfish (*Tetraodon nigroviridis*; [GenBank:CAAE01014252], medaka (*Oryzias latipes*; located in scaffold16794) and grouper [GenBank:AY735011] were also identified by database search for comparative analysis. Interestingly, we were unable to identify any HIF-3α-like isoforms in fish. Likewise, *in silico *analysis of the genome sequences of a number of mammalian species (human, mouse, rat, cow, dog and cat) in the GenBank database failed to turn up any HIF-4α-like sequences.

**Figure 3:**
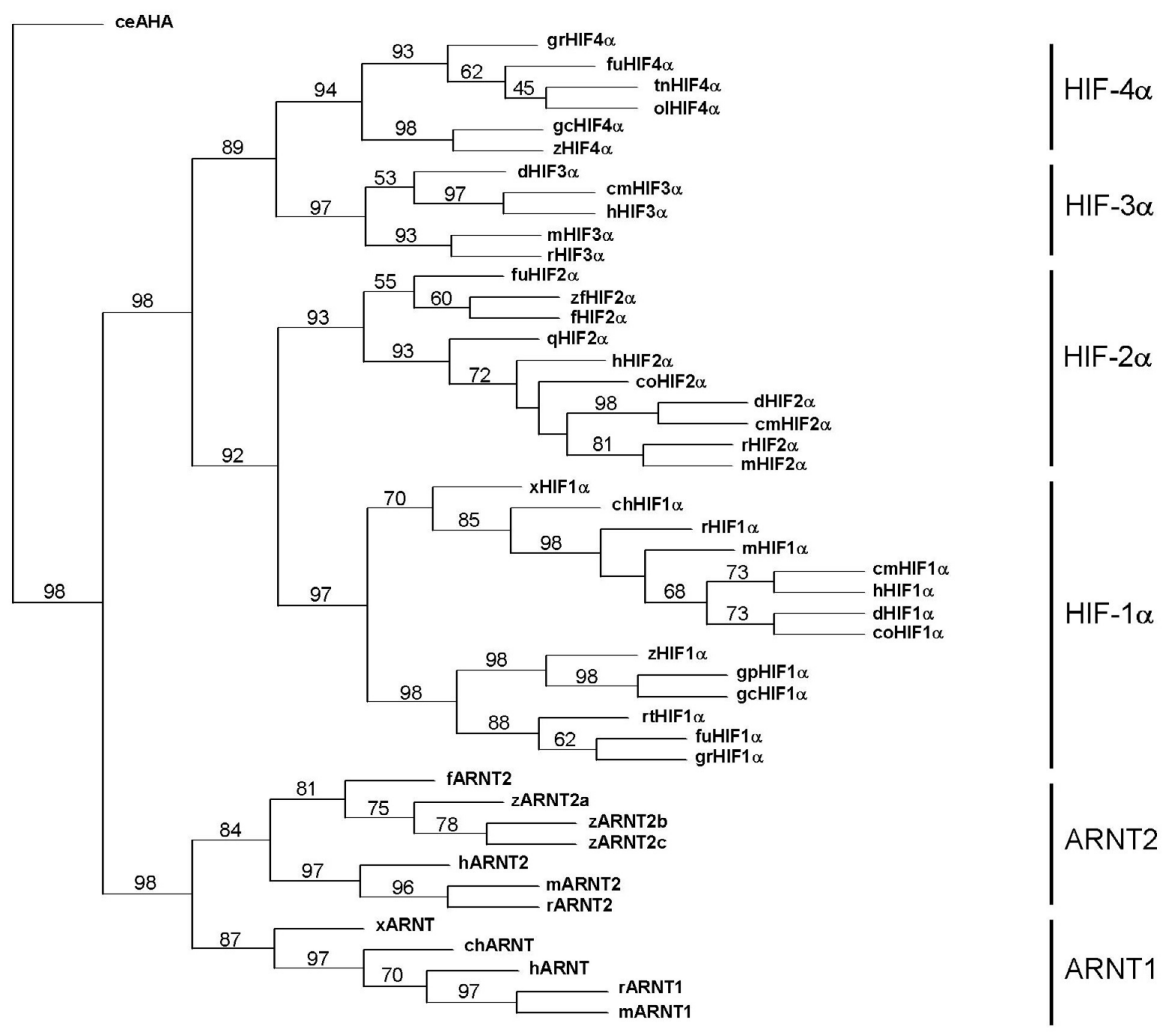
**Phylogenetic analysis**. Phylogenetic analysis of gcHIF1α and gcHIF4α against selected bHLH-PAS proteins from different animals. Phylogeny was performed on bHLH-PAS sequences by maximum parsimony (PROTPARS) using the PHYLIP package version 3.57 c [25]. The bootstrap support (SEQBOOT program, PHYLIP package) for each branch (1000 replications) is shown. The standing of the abbreviations and the GenBank/EMBL/Swissprot accession number of the bHLH-PAS-A/B sequences used are provided in additional file 1.

Because the different HIF-α subtypes share high sequence conservation in the N-terminal bHLH-PAS(A/B) domain but are highly divergent in the C-terminal region, only the bHLH-PAS(A/B) sequences of the HIF-α s, ARNT1 and ARNT2 proteins from various vertebrate species were multiply aligned for phylogenetic analysis. Using the *Caenorhabditis elegans *AHA protein as outgroup, a maximum parsimony tree was constructed (bootstrapped with 1000 replications) using the PHYLIP package [25]. As shown in Fig. 3, two closely-related subclades of HIF-1α s (97% bootstrap support) – one consisting of avian, *Xenopus *and mammalian HIF-1α s and the second consisting exclusively of fish HIF-1α homologues – were obtained. As expected, the HIF-1α clade is shown to be more closely related to the HIF-2α (92% bootstrap support) than the HIF-3α and HIF-4α clades. Most intriguingly, the putative HIF-4α proteins from six different fish species formed a distinct clade (94% bootstrap support) which is phylogenetically more similar to the mammalian HIF-3α s (89% bootstrap support) than to other HIF-α proteins. Phylogenetic analysis using the neighbor-joining method also produced a tree of the same topology with similarly high bootstrap scores (data not shown).

### In vivo expression and response patterns of gcHIF-1α and gcHIF-4α to short- and long-term hypoxia

**Figure 4:**
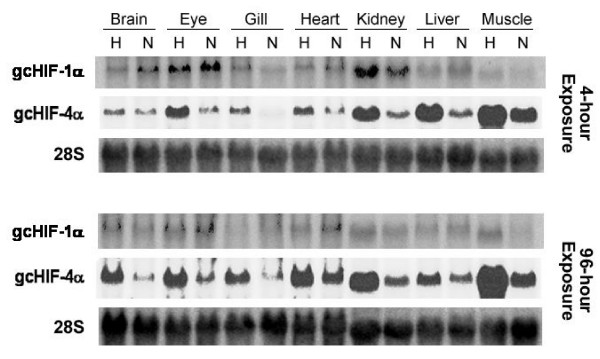
**Northern blot analysis of gcHIF-1α and gcHIF-4α **. A representative Northern blot derived from the tissues of one normoxic and one hypoxic fish from a total of five in each group is shown. Total RNA (20 μg) samples from various tissues of grass carp exposed to normoxia (N) and hypoxia (H) for 4 and 96 h were analysed by Northern hybridization using SL18 (gcHIF1α), SL12 (gcHIF4α) and a 115-bp grass carp 28S rDNA fragment as probes.

To examine the *in vivo *expression and response patterns of *gcHIF-1α *and *gcHIF-4α *to hypoxia, grass carps were exposed to normoxic (7 mg O_2_/l) and hypoxic (0.5 mg O_2_/l) conditions and fish (n = 5) were sampled from each treatment group and control after 4 and 96 h. Total RNA was isolated from seven different tissues of each of five fish from the normoxic and hypoxic groups at each time point for Northern blot analysis. Overall, normoxic expression and hypoxic induction patterns of the *gcHIF-1α *and *gcHIF-4α *genes were consistent amongst the replicate blots derived from different fish tissues, and a representative autoradiogram is shown in Fig. 4. Under normoxic conditions, the 3.9-kb *gcHIF-1α *mRNA transcript was expressed most abundantly in eye and kidney; with lower expression levels being detected in brain, gill, heart and liver; and negligible expression in muscle. In contrast, the 3.7-kb *gcHIF-4α *transcript was expressed at comparatively higher levels in brain, heart, kidney, liver and muscle relative to *gcHIF-1α *under similar conditions. A marked increase in *gcHIF-1α *expression was observed in gill and kidney after exposure to hypoxia for 4 h (but not at 96 h); while *gcHIF-1α *expression was seemingly downregulated in brain, heart and liver, and appeared unchanged in eye. This is in sharp contrast to observations in rainbow trout [12] and mammals [26-28] where HIF-1α mRNA levels are unaffected by hypoxia. Interestingly, *gcHIF-4α *(the larger 3.7-kb transcript) was markedly upregulated following exposure to hypoxia for 4 and 96 h in eye, gill, heart, kidney, liver and muscle. Curiously, although the less abundant 2.1-kb *gcHIF-4α *transcript showed prominent expression and hypoxic up-regulation (ca. 5-fold) in kidney; expression of this smaller *gcHIF-4α *transcript was barely detectable in all other tissues examined under both normoxic and hypoxic conditions (data not shown).

Expression levels of *gcHIF-1α *and *gcHIF-4α *in all replicates of each hypoxic tissue were normalized against 28S rRNA and compared with the normoxic counterpart. If expression levels were the same in both the hypoxia treatment and normoxia control groups, the hypoxia:normoxia expression ratio should theoretically be equal to 1; whereas the ratio is expected to be significantly greater than 1 if hypoxic induction of the genes was observed. The 4- and 96-h datasets of each tissue were lumped and a non-parametric χ^2 ^test was performed to test the null hypothesis that the hypoxia:normoxia expression ratio was not significantly different from 1 [29]. The analysis indicated that while *gcHIF-1α *expression was significantly higher in hypoxic kidney as compared to the normoxic control (*P *< 0.05), hypoxic induction of *gcHIF-4α *was found to be comparatively higher in eye, gill, heart, kidney and liver (*P *< 0.05).

### Western blot analysis of gcHIF-1α and gcHIF-4α

**Figure 5:**
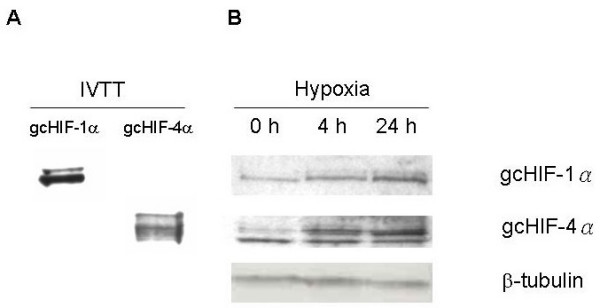
**Western blot analysis of gcHIF-1α and gcHIF-4α proteins in hypoxic grass carp liver**. (A) gcHIF-1α and gcHIF-4α proteins were synthesized by *in vitro *transcription and translation (IVTT) in *E. coli *lysate (Roche) and used as positive controls to test the specificity of the rabbit anti-gcHIF-1α (AB-1) and anti-gcHIF-4α (AB-4) polyclonal antibodies. No cross reactivity was detected with either of these antibodies. (B) Liver extracts were prepared from grass carps exposed to hypoxia (DO 0.5 ± 0.3 mg/l) for 0, 4 and 24 h and total protein (30 μg each) analysed by Western blotting for gcHIF-1α and gcHIF-4α using polyclonal antibodies AB-1 and AB-4, respectively. Signals were normalized against the β-tubulin protein. A representative Western blot from three different fish (n = 3) is shown.

To investigate the endogenous gcHIF-1α and gcHIF-4α protein levels in grass carp liver in normoxia and hypoxia, liver extracts from fish exposed to 0, 4 and 24 h of hypoxia were analysed by Western blotting using gcHIF-1α – (AB-1) and gcHIF-4α-specific (AB-4) rabbit antisera. The specificity of these polyclonal antibodies was confirmed using *in-vitro*-transcribed and -translated gcHIF-1α and gcHIF-4α proteins which were detected as 86-kDa and 72-kDa protein bands, respectively (Fig. 5A). No cross reactivity was observed with the AB-1 and AB-4 antibodies on gcHIF-4α and gcHIF-1α, respectively (data not shown).

As shown in Fig. 5B, the AB-1 and AB-4 antibodies each detected at least two protein bands in the fish liver extracts, which most likely correspond to post-translationally-modified (differentially phosphorylated) forms of the respective gcHIF-α proteins. Interestingly, gcHIF-1α is clearly detectable in the liver of normoxic grass carp (0 h hypoxia exposure) and is increased by ca. 1.5- and 2.5-fold, respectively, following exposure to 4 h and 24 h of hypoxia. This is in striking contrast to previous observations in mouse [30,31] and rainbow trout [12] where HIF-1α is not detectable in liver (or hepatocytes) under normoxic conditions due to rapid proteasomal degradation of the protein. As for the gcHIF-4α protein, its expression was also evident in grass carp liver under normoxic conditions, and showed a steady increase following exposure to 4 h (ca. 2.5-fold) and 24 h (ca. 3-fold) of hypoxia.

### Induction of p(HRE)4-LUC reporter expression by gcHIF-1α and gcHIF-4α in CHO cells

**Figure 6:**
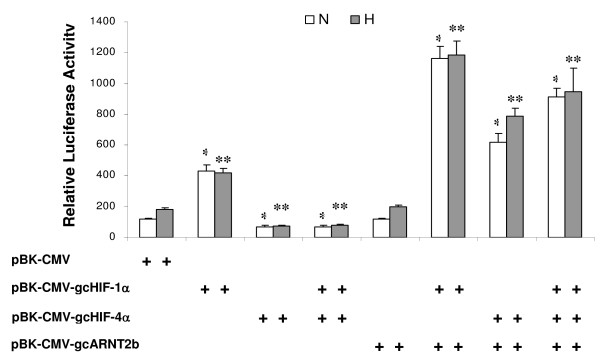
**Effects of gcHIF-1α and gcHIF-4α on HRE-Luc reporter activity in CHO cells**. CHO-K1 C4.5 cells were cotransfected with pBK-CMV-gcHIF-1α, pBK-CMV-gcHIF-4α or pBK-CMV empty vector (with or without pBK-CMV-gcARNT2b) along with the p(HRE)_4_-Luc reporter and pSVβ-galactosidase plasmids. Transfected cells were exposed to normoxia (N, open bars) or hypoxia (H, shaded bars) for 16 h and then assayed for luciferase and β-galactosidase activities. The constituent plasmid(s) in each transfection experiment is/are indicated by a + sign underneath the respective bar charts. Luciferase was normalized against β-gal activity and data represent the mean ± SEM of 5 independent experiments. *, *P *< 0.05 between the gcHIF-α – and empty vector-transfected groups under normoxia; **, *P *< 0.05 between the gcHIF-α – and empty vector-transfected groups under hypoxia.

To compare the transcriptional activity of gcHIF-1α and gcHIF-4α, CHO-K1 C4.5 cells were cotransfected with a luciferase reporter plasmid containing the human EPO HRE element together with expression vectors for either gcHIF-1α or gcHIF-4α followed by exposure to normoxia or hypoxia. As shown in Fig. 6, luciferase activity in cells transfected with the pBK-CMVgcHIF-1α expression vector showed ca. 2.5-fold increase while cells transfected with pBK-CMVgcHIF-4α showed a 50% decrease in activity, respectively, when compared to cells transfected with the empty pBK-CMV vector (*P *< 0.05). Interestingly, coexpression of pBK-CMVgcHIF-4α markedly reduced the transcriptional activity of pBK-CMVgcHIF-1α by more than 5-fold (*P *< 0.05), suggesting that gcHIF-1α may be competitively inhibited by gcHIF-4α. To examine whether the reduction in gcHIF-1 activity by gcHIF-4α may be attributed to limiting amounts of endogenous ARNT; a gcARNT2b expression plasmid was cotransfected into CHO cells together with pBK-CMVgcHIF-1α and/or pBK-CMVgcHIF-4α. While the transfection of gcARNT2b alone showed no stimulatory effect on the HRE-luciferase reporter gene, cells cotransfected with gcARNT2b and gcHIF-1α or gcHIF-4α showed ca. 7-fold and 5-fold increase in luciferase activity, respectively (*P *< 0.05), relative to the empty vector. Overall, the induction of luciferase activity was consistently higher in the gcHIF-1α – than gcHIF-4α-transfected cells (Fig. 6). Cells cotransfected with gcHIF-1α, gcHIF-4α and gcARNT2b also showed a marked increase in luciferase activity as compared to the empty vector control (*P *< 0.05). Overall, no significant difference in luciferase activity was observed between normoxic and hypoxic gcHIF-α-transfected cells.

## Discussion

We have identified and characterized the expression pattern and transcriptional activity of two distinct HIF-α isoforms – gcHIF-1α and gcHIF-4α – from the grass carp. To our knowledge, the present study is the first to report the cloning and comparative analysis of two HIF-α isoforms from a hypoxia-tolerant fish species. The open reading frames (ORFs) of gcHIF-1α and gcHIF-4α are 2325 bp and 1932 bp in length, and encode proteins of 774 and 643 amino acids, respectively. High sequence conservation is observed in the N-terminal portion of these two proteins which contain all of the characteristic motifs typical of HIF-α proteins including: the basic helix-loop-helix (bHLH) domain; N-terminal nuclear localization signal (N-NLS); Per-Sim-ARNT (PAS)-A and -B domains; PAS-associated C-terminal (PAC) domain; oxygen-dependent degradation (ODD) domain; and N-terminal transactivation (N-TAD) domain (Figs. 1 and 2). Sequence comparison showed that while gcHIF-1α is most similar to known HIF-1α s (57–68%) from different vertebrate species, gcHIF-4α showed an equivalent degree (41–47%) of sequence identity in the N-terminal bHLH and PAS domains (but not the C-terminal domain) with the HIF-1α, HIF-2α and HIF-3α proteins so far described, which led us to believe that gcHIF-4α is a novel HIF-α isoform.

Although the C-terminal region of gcHIF-4α is poorly conserved in comparison with other vertebrate HIF-α s (Fig. 2), the fact that it is capable of activating HRE-driven transcription of the luciferase gene (Fig. 6) suggests that it probably contains an atypical transactivation domain with properties distinct from that of the CTADs of HIF-1α, -2α and -3α. Traditionally, transcription factors have been classified according to the prevalence of certain amino acids in the activation domains such as glutamine-rich, proline-rich and acidic-classes [32]. Analysis of the C-terminal region of gcHIF-4α (amino acids 525–643; Fig. 2) showed that it is significantly more acidic (20% are aspartic acid and glutamic acid residues) than that of HIF-1α (12–13%), HIF-2α (12–14%) and HIF-3α (8–11%), which suggests that gcHIF-4α likely contains an acidic activation domain. However, further experiments are needed to further characterize and map the functional activation domain of this region.

Using Northern hybridization, we demonstrated that gcHIF-1α is expressed as a single 3.8-kb transcript while gcHIF-4α is expressed in two forms – a major 3.1-kb transcript and a minor 2.1-kb transcript. We speculated that the two gcHIF-4α mRNA transcripts are generated either through the use of alternative transcriptional start sites or polyadenylation sites although numerous attempts at using 5' – and 3' -RACE, and degenerate RT-PCR to identify an alternatively spliced variant(s) of gcHIF-4α (in addition to clone SL12; Fig. 1) have been unsuccessful. Nonetheless, it was demonstrated that the major 3.1-kb gcHIF-4α transcript is ubiquitously expressed in a wide variety of fish tissues in normoxia, albeit at varying levels, and differs from gcHIF-1α which exhibited a more restricted expression profile (Fig. 4). Additionally, the response pattern to hypoxia also differs substantially between the two genes whereby gcHIF-4α expression is markedly upregulated in eye, gill, heart, kidney, liver and white muscle following exposure to short- (4 h) and long-term (96 h) hypoxia, while gcHIF-1α expression was only marginally upregulated in gill and kidney at 4 h (but not 96 h) hypoxia (Fig. 4). These findings indicated that gcHIF-1α expression and its regulation is markedly different from its counterparts in hypoxia-sensitive animal species such as rainbow trout [12], mouse, rat and human [26-28,31] where HIF-1α is ubiquitously expressed and at a constant level under normoxic and hypoxic conditions. On the other hand, increases in endogenous gcHIF-1α protein levels in liver of hypoxic fish (Fig. 5) suggest that gHIF-1α may be stabilized (or its translation increased) under hypoxic conditions in a manner similar to mammalian HIF-1α [3]. Overall, the differential expression and regulation patterns of gcHIF-1α and gcHIF-4α at the mRNA and protein levels indicate that both genes play very specialized roles in different fish organs in normoxia and are essential for tissue response to short- (4 h) and long-term (96 h) hypoxia in grass carp.

To explore whether a HIF-4α subtype exist in mammals, TBLASTN search of the genome sequences and EST databases of all available mammalian species (at NCBI) was performed and a number of hits (which showed between 33–49% sequence similarity to the bHLH and PAS domains of gcHIF-4α) were obtained. However, closer inspection indicated that the clones encode for different members of the bHLH-PAS superfamily of proteins such as HIF-1α, HIF-2α, HIF-3α, ARNT, AhR, CLOCK, SIM1, SIM2 and various MOP isoforms, and none showed any significant similarity to the C-terminal region of gcHIF-4α. Based on this, we believe that HIF-4α is probably not found in mammals and highly likely a novel isoform specific to fish. Conversely, HIF-3α has thus far only been reported in mammals and bioinformatic searches of several fish (Japanese pufferfish, green puffer, zebrafish and medaka) genome and EST databases (NCBI) also failed to turn up any HIF-3α-like sequence, which suggest that HIF-3α may not be present in the genomes of fish. Interestingly, parsimony analysis of 35 HIF-α and 13 ARNT proteins from different animal species indicated that the putative HIF-4α s from fish form a distinct clade that is phylogenetically more closely related to the mammalian HIF-3α s (89% bootstrap support) than to either the HIF-1α or HIF-2α proteins (Fig. 3). This raises the intriguing possibility that HIF-3α and HIF-4α are probably orthologs that may have arisen following lineage divergence between mammals and fish some 350–400 million years ago [33].

There are a number of similarities (at the expression and functional levels) between gcHIF-4α and mammalian HIF-3α which are in line with this notion. First, expression of gcHIF-4α (Fig. 4) and mammalian HIF-3α [28] is not only evident under normoxic conditions in tissues such as brain, heart, kidney and liver, but the mRNA levels are also markedly upregulated in these same tissues in fish and rat, respectively, in response to hypoxia. In a related series of experiments, we have observed (by Northern hybridization) that *VEGF-A *mRNA levels in grass carp are also significantly upregulated in brain, heart, kidney and liver tissues upon exposure to hypoxia for 4 h which indicated that the HIF system has indeed been activated under these conditions (unpublished observations). Most intriguingly, the increase in *VEGF-A *mRNA levels correlated with the hypoxic increase in gcHIF-4α (but not gcHIF-1α) mRNA levels in the same tissues (Fig. 4). These results are consistent with the notion that the function of gcHIF-4α, and the mechanism(s) regulating its expression in grass carp, may be similar to that of HIF-3α in mammals, which purportedly represents a rapidly responding HIF system to short-term tissue hypoxia [28].

Independent evidence supporting the functional similarity of gcHIF-4α to HIF-3α is provided by HRE-luciferase reporter gene activation studies in which overexpression of gcHIF-4α was found to attenuate the transcriptional activity of gcHIF-1α in CHO cells (Fig. 6); a phenomenon analogous to the suppressive effect of HIF-3α on HIF-1 activity in mammalian cells [34]. Whether the suppression by gcHIF-4α is due to its competition with gcHIF-1α for ARNT or other bHLH-PAS dimerization partners such as CLIF [35] and MOP [36]; or its direct binding to HIF-1α similar to the inhibitory PAS (IPAS) protein which prevents the nuclear import of HIF-1α and its subsequent binding to the HREs of downstream target genes [37], is presently not known. On the other hand, it was observed that when cotransfected with ARNT, gcHIF-4α (Fig. 6) and mammalian HIF-3α [34,38] are each able to activate HRE-driven transcription, indicating that both are capable of operating as weak transcription factors. These observations suggest that gcHIF-4α, similar to HIF-3α, may play a dual regulatory role as a transcriptional activator (HIF-ARNT dimer) or repressor (presumably in its monomeric form), depending on the availability of compatible ARNT molecules (Fig. 6).

It is recognized that the enhancement of transcriptional activation is dependent upon the stability and translocation of specific HIF-α proteins from the cytoplasm to the nucleus and their subsequent binding to the HREs of target genes [3]; and a key factor modulating these processes is the intracellular redox state [39-41]. Recently, it has been shown that the stability and DNA-binding activity of rainbow trout HIF-1α (rtHIF-1α) is regulated by a redox mechanism [41] very similar to the mammalian HIF-2α [40], whereby the redox sensitivity of both proteins was attributed to the Cys28 residue in the rtHIF-1α (Cys25 in mammalian HIF-2α) protein. Changing the Ser28 residue to Cys28 by site-directed mutagenesis in mammalian HIF-1α rendered its DNA-binding activity redox sensitive [40]. Interestingly, the amino acid at position 28 of gcHIF-1α and gcHIF-4α are, respectively, Ser27 and Cys24 (Fig. 2), suggesting that the stability and DNA-binding activity of both proteins may be differentially sensitive to redox states. Conceivably, redox regulation through sulfhydryl modifications of cysteine residues could also potentially occur in the C-terminal transactivation domain, wherein the redox-sensitive residues may affect both the stability and activity of the protein [41]. Whether these aspects of gcHIF-1α and gcHIF-4α are regulated by cellular redox states in the grass carp will need to be elucidated in future studies.

## Conclusion

We have demonstrated that there are at least two distinct HIF-α isoforms – gcHIF-1α and gcHIF-4α – in the hypoxia-tolerant grass carp and that they are differentially expressed and regulated in grass carp in response to hypoxic stress in different fish organs. Our findings suggest that unique molecular mechanisms operate through these two HIF-α isoforms, which underpin the hypoxic response in the hypoxia-tolerant grass carp. However, the *in vivo *functions of gcHIF-1α and gcHIF-4α remains to be elucidated, and experiments are underway in our laboratory to compare their stability, nucleocytoplasmic distribution and DNA binding specificity to the HREs of hypoxia-inducible genes such as *gcGlutX *[42], *gcCITED *[43] and *gcVEGF-A *[44] in grass carp using the chromatin immunoprecipitation and electrophoretic mobility shift assays. In addition, a comparison of gcHIF-4α with the corresponding orthologues in hypoxia-sensitive fish species such as rainbow trout would provide further insights into the role(s) of this novel isoform in hypoxia tolerance mechanisms in fish.

The full-length cDNA sequences of gcHIF-1α and gcHIF-4α have been deposited in the GenBank database under accession numbers [GenBank:AY450269 and AY450270], respectively.

## Methods

### Fish

Animal care and experiments were undertaken in accordance with City University of Hong Kong animal care guidelines. Grass carp, *Ctenopharyngodon idellus *(body weight of around 500 g) were obtained from a commercial hatchery and acclimatized in 300-l fiberglass tanks with circulating, filtered and well-aerated tap water at 20 ± 1°C for 1 week prior to experimentation. Fish were reared under normoxia (7.0 ± 0.2 mg O_2_/l) or hypoxia (0.5 ± 0.3 mg O_2_/l) for 4 and 96 h in a continuous flow system as previously described by Zhang et al [42]. Dissolved oxygen was monitored continuously using an YSI Model 580 dissolved oxygen meter (Geo Scientific Inc.). After the exposure period, fish were anaesthetized and tissues were immediately dissected out, snap-frozen in liquid nitrogen and stored at -80°C until ready to be processed.

### Cell cultures and treatments

CHO-K1 cells, a gift from Professor Peter Ratcliffe (Wellcome Trust Center for Human Genetics, Oxford University, UK), were cultured in DMEM supplemented with 10% fetal calf serum (Invitrogen)), 0.1 mM non-essential amino acids (Invitrogen) and antibiotics (penicillin G, 100 U/ml; streptomycin, 100 μg/ml) (Invitrogen), in humidified air containing 5% CO_2 _at 37°C. Cells were exposed to either normoxia (20% O_2_) or hypoxia (1% O_2_) by incubating them in an anaerobic incubator (NUAIRE) in 5% CO_2_, 94% N_2 _at 37°C.

### RNA isolation and cloning of full-length cDNAs

Total RNA was extracted from grass carp tissues using the Trizol reagent (Invitrogen) according to the manufacturer's instructions. Poly (A)^+ ^RNA was purified from total RNA using the PolyATract System kit (Promega). Using different combinations of degenerate primers, HIF-F1 (AACATMAAGTCTGCHACRTGGAAGGT), HIF-F2 (TGYGAACCHATWCCTCAYCCATC), HIF-R2 (GRGATR TANGGDGCYARCATBTCCAA) and HIF-R3 (TCAGTTRACTTGRTCCARR GCWCT) which were designed against consensus sequences derived from multiple alignment of human [GenBank:AF208487], bovine [GenBank:AB018398], mouse [GenBank:NM_010431], rat [GenBank:Y09507] and rainbow trout [GenBank:AF304864] HIF-1α cDNA sequences, reverse transcription PCR (RT-PCR) with total RNA from grass carp kidney was performed. PCR in a 100- μl mixture was performed on first-strand cDNAs that were reverse transcribed from total RNA (1 μg) using Superscript II RNase H^- ^reverse transcriptase (Invitrogen) with oligo-dT_12–18 _as primers and consisted of 20 ng of first strand cDNA, 1 × PCR buffer (20 mM Tris/HCl pH 8.4, 50 mM KCl), 1 μM of each primer, 0.2 mM of dNTPs, 1.5 mM MgCl_2 _and 5 U of Platinum *Taq *DNA polymerase (Invitrogen). The PCR program consisted of predenaturation at 94°C for 3 min, followed by 35 cycles of amplification (denaturation at 94°C for 1 min, annealing at 55°C for 1 min, and extension at 72°C for 2 min) and a final extension at 72°C for 10 min in a Gene Cycler (Bio-Rad, USA). A 0.45-kb RT-PCR product (designated as c12) was obtained with primer pair, HIF-F1 and HIF-R2, and a 0.8-kb RT-PCR product (designated as c18) was obtained with primer pair, HIF-F1 and HIF-R3. DNA sequencing indicated that both cDNA fragments encode for peptides that share significant sequence similarity to HIF-α proteins. To obtain the full-length cDNAs of c12 and c18, 5' – and 3' -RACE PCR were performed using the SMART RACE cDNA Amplification kit (Clontech). Poly (A)^+ ^RNA (1 μg) from kidney of hypoxically-stressed grass carp was used as a source of template. Gene-specific nested primers for 5' -RACE were: 1α-5'GSP1 (ACGGCTAAGGAAGGTCTTGCTGTCC) and 1α-5'GSP2 (CCTCGTTGTTTGAGGGATGAGGAATGG) for c18; and 4α-5'GSP1 (TATCCAACAAGCTCAGTGACTCGTC) and 4α-5'GSP2 (GTGAGGAAAGTGCTGCTGTCAAGTG) for c12. Gene-specific nested primers for 3' -RACE were: 1α-3' GSP1 (GGTGCTTTGCTTCAGAGTGTGGACAG) and 1α-3' GSP2 (GAAGTTAAAGGATCGAGTGTGCTCGG) for c18; and 4α-3' GSP1 (CAGCTTCCTGACATTGCTGTGTGAG) and 4α-3' GSP2 (GACGAGTCACTGAGCTTGTTGGATA) for c12. Thirty-five cycles of PCR were performed according to the manufacturer's recommendations. RACE products were cloned into the pGEM-T Easy vector (Promega) for DNA sequencing. Full-length cDNAs were obtained by RT-PCR using two pairs of gene-specific primers: 1α-F (TACCGACTAGAAGCTGCACCGA) and 1α-R (AGAAAGACTGGAGACTGCAG AGA) for c18; and 4α-F (GATAAACAAGCTGCAGACGACGT) and 4α-R (GGCA TGGTTTCAGCAACAGGT) for c12.

### Northern blot analysis

Total RNA (20 μg) was electrophoresed on 1% (w/v) agarose/formaldehyde gel and blotted onto Hybond-XL membrane (Amersham Biosciences). Blots were prehybridised at 65°C for 30 min in ExpressHyb solution (Clontech) and Northern hybridization carried out at 65°C for 2 h in the same solution containing 2.0 × 10^6 ^cpm/ml of [^32^P]dCTP-labelled cDNA probe prepared by random priming (GE Healthcare). Blots were washed thrice in 2 × standard sodium citrate (SSC), 0.05% (w/v) sodium dodecyl sulphate (SDS) for 10 min at room temperature, and twice in 0.1 × SSC, 0.1% SDS (w/v) for 20 min at 50°C. Blots were exposed on a phosphor screen (Kodak-K) at room temperature for 20 h, and the signals were captured and quantified using the Molecular Imager FX System (Bio-Rad). A 115-bp 28S rDNA fragment (and confirmed by DNA sequencing) was amplified from grass carp total DNA using primers 28S-F (GATCCTTCGATGTCGGCTCT) and 28S-R (CTAACCTGTCTCACGACGGT) and used as an internal control probe in northern hybridization for normalization of gene expression.

### Plasmid construction

Coding regions of SL18 (gcHIF-1α) and SL12 (gcHIF-4α) were PCR amplified, respectively, using primers c18F (ATCCACATATGGATACTGGAGTTGTCACTG; *Nde*I site is underlined) and C18R (ATTACCCGGGGTTGACTTGGTCCAGAGCACG; *Sma*I site is underlined); and primers c12F (ATCCACATATGGTGAACTCGGTGAAT; *Nde*I site is underlined) and c12R (ATTACCCGGGAGTAAGGGACGCGAATTCACTAGTG; *Sma*I site is underlined). PCR fragments were double digested with *Nde*I/*Sma*I and subcloned into the pIVEX 2.3-MCS prokaryotic expression vector (Roche) to produce pIVT-gcHIF-1α and pIVT-gcHIF-4α plasmids. *In vitro *expression of the gcHIF-1α and gcHIF-4α cDNAs was performed using the Rapid Translation System (RTS 100) kit (Roche). Expression vector pBK-CMV-gcHIF-1α was generated by RT-PCR amplification of gcHIF-1α using primers HIF-1α-F (TAATAGGCTAGCCTCGCGGTTTGGAAAAACCTAACAC; *Nhe*I site is underlined) and HIF-1α-R (TCATGCTCGAGAGTTGACTTGGTCCAGAGCACG; *Xho*I site is underlined) to produce a 2.35-kb gcHIF-1α cDNA fragment which was subcloned into the *Nhe*I/*Xho*I sites of pBK-CMV (Stratagene). Expression vector pBK-CMV-gcHIF-4α was generated by RT-PCR amplification of gcHIF-4α using primers HIF-4α-F (ATCCAGGATCCGAATTTGCAGTGATTTCACTAG; *Bam*HI site is underlined) and HIF-4α-R (TCATGCTCGAGTAAGGGACGCGAATTCACT; *Xho*I site is underlined) to produce a 1.95-kb gcHIF-4α cDNA fragment which was subcloned into the *Bam*HI/*Xho*I sites of pBK-CMV. Expression vector pBK-CMV-gcARNT2b was generated by RT-PCR amplification of gcARNT2b [GenBank:AY596921] using primers gcARNT2b-F (TTGCTAGCTAACCGAGGCAATATGGCAACACC; *Nhe*I site is underlined) and gcARNT2b-R (CACTCGAGTCTCCGGTTACTCAG; *Xho*I site is underlined) to produce a 2.2-kb gcARNT2b cDNA fragment which was subcloned into the *Nhe*I/*Xho*I sites of pBK-CMV. All constructs were verified by DNA sequencing.

### Transient transfection and luciferase assays

CHO cells were seeded in 24-well plates at 1 × 10^5 ^cells per well and transfected with 120 ng of p(HRE)_4_-Luc reporter plasmid and 60 ng of pSVβ-Galactosidase expression vector (Promega, Madison, WI) using LipofectAMINE 2000^® ^transfection reagent (Invitrogen) in OptiMEM^® ^I reduced serum medium according to the manufacturer's instructions. The p(HRE)_4_-Luc reporter plasmid consists of 4 copies of the human erythropoietin hypoxia-responsive element (HRE) linked to the SV40 promoter and firefly luciferase gene (a gift from Professor Yoshiaki Fujii-Kuriyama, Center for Tsukuba Advanced Research Alliance and Institute of Basic Medical Sciences, University of Tsukuba, Japan). Ectopic expression of gcHIFα involved cotransfection of 120 ng of the respective gcHIF-α expression plasmid(s) or an equimolar amount of the empty pBK-CMV vector with the luciferase and β-Gal reporter plasmids in the presence/absence of 120 ng of pBK-CMV-gcARNT2b. Transfected cultures were incubated in fresh medium at 37°C for 24 h and then exposed to normoxia (21% O_2_) or hypoxia (1% O_2_) for 16 h. Luciferase activities of cell lysates were determined using the Bright-Glo™ Luciferase assay kit (Promega, Madison, WI) according to the manufacturer's instructions and normalized against theβ-galactosidase values to correct for variations in transfection efficiency. Data given are means ± S.E.M. from five independent experiments with three replicates per sample in each experiment.

### Protein extraction and immunoblot analysis

Fish tissues were homogenized using a Polytron tissue disruptor (Kinematica AG) in protein extraction buffer (420 mM NaCl, 1.5 mM MgCl_2_, 0.2 mM EDTA, 20 mM HEPES) with protease inhibitors (0.5 mM dithiothreitol, 0.4 mM phenylmethylsulfonyl fluoride, 2 μg/ml each of leupeptin, pepstatin A and aprotinin; Sigma). Protein concentration was determined using the Bradford assay (BioRad). Anti-gcHIF-1α (AB-1) and anti-gcHIF-4α (AB-4) polyclonal antibodies were raised in different rabbits, respectively, using gcHIF-1α-specific peptides corresponding to amino acids 153–167 [CSKKTKEQNTERSFFL] and amino acids 630–644 [CPFSGSRDTSPARSPT], and gcHIF-4α-specific peptides corresponding to amino acids 77–91 [CTKTEETENPTDGFYQ] and amino acids 576–591 [SDDASEEFEPPPQKRC] (Fig. 2); and were custom produced by Eurogentec (Bruxelles, Belgium). Total proteins were separated in 10% SDS PAGE, transferred to nitrocellulose membranes (Millipore, Bedford, MA), blocked in 4% non-fat dry milk and incubated with AB-1 (1:100 dilution), AB-4 (1:100 dilution) or rabbit anti-β-tubulin (1:1000; Santa Cruz, CA) antibodies, followed by detection with HRP-conjugated secondary anti-rabbit IgG antibody (1:5000 dilution in 1% BSA in PBS) using the ECL Western-blotting system (GE Healthcare). Membranes were exposed to BioMax MR films (Kodak) and relative intensities of bands were estimated using the Quantity 1 software (BioRad, U.S.A.).

### Phylogenetic analysis

Phylogenetic analysis was performed by maximum parsimony using the PROTPARS program of the PHYLIP package version 3.57 c [25]. Support for the inferred clades was obtained by bootstrap analysis from 1000 replications of the data set using the SEQBOOT and CONSENSE programs. Phylogenetic tree was displayed using TREEVIEW [45]. Sequence analyses and homology searches were performed using the online BLAST suite of programs (NCBI, USA).

### Statistical analysis

A non-parametric χ^2 ^test was used to test the null hypothesis that the ratio of hypoxic:normoxic mRNA expression level was not significantly different from 1 [29]. One-way ANOVA was used to examine effects of ectopic expression of different gcHIF-α s on HRE-driven luciferase activity. Where significant effects were detected, Tukey's tests were performed to identify significant difference between individual means; α = 0.05 was used in all statistical tests.

## Abbreviations

ARNT, aryl hydrocarbon receptor nuclear translocator; HIF, hypoxia-inducible factor; FIH, factor inhibiting HIF-1; CHO, Chinese hamster ovary; EST, expressed sequence tag; PHD, prolyl hydroxylase.

## Authors' contributions

SHWL carried out most of the experimental work described in this paper and drafted the manuscript. PKSN and RMKY assisted with many of the expression studies. RSSW and RYCK contributed to the design and planning of this study and edited the manuscript.

## Supplementary Material

Additional File 1 **Standing of the abbreviation and GenBank/EMBL/Swissprot accession number of the bHLH-PAS-A/B sequences used in Fig. **3. The abbreviations are listed from top to bottom in accordance with Fig. 3 – Phylogenetic analysis. Click here for file
