# Supplementary material for: Cloning and expression analysis of two distinct HIF-alpha isoforms – gcHIF-1alpha and gcHIF-4alpha – from the hypoxia-tolerant grass carp, Ctenopharyngodon idellus
Source: BMC Mol Biol. 2006 Apr 20;7:15. doi: 10.1186/1471-2199-7-15 (PMC1473195; doi:10.1186/1471-2199-7-15)
Supplement: Additional File 1 — Standing of the abbreviation and GenBank/EMBL/Swissprot accession number of the bHLH-PAS-A/B sequences used in Fig. 3. The abbreviations are listed from top to bottom in accordance with Fig. 3 – Phylogenetic analysis. [file 1471-2199-7-15-S1.doc]

| **Abbreviations** | **Standings** | **Accession numbers** |
| --- | --- | --- |
| ceAHA | *Caenorrhabditis elegans* ARNT | GenBank:AAB99999 |
| grHIF4α | grouper HIF-4α | GenBank:AY735011 |
| fuHIF4α | *Fugu* HIF-4α | Scafffold266 |
| tnHIF4α | *Tetraodon nigroviridis* HIF-4α | GenBank:CAAE01014252 |
| olHIF4α | *Oryzias latipes* HIF-4α | Scaffold16794 |
| gcHIF4α | grass carp HIF-4α | GenBank:AY450270 |
| zHIF4α | zebrafish HIF-4α | GenBank:AY330295 |
| dHIF3α | dog HIF-3α | GenBank:XM_533636 |
| cmHIF3α | chimpanzee HIF-3α | GenBank:XP_512767 |
| hHIF3α | human HIF-3α | GenBank:AAD22668 |
| mHIF3α | mouse HIF3α | GenBank:AAC72734 |
| rHIF3α | rat HIF-3α | GenBank:CAB96611 |
| fuHIF2α | *Fugu* HIF-2α | Scaffold159 |
| zHIF2α | zebrafish HIF-2α | GenBank:BX248102 |
| fHIF2α | *Fundulus* HIF-2α | GenBank:AAL95711 |
| qHIF2α | quail HIF-2α | GenBank:AAF21052 |
| hHIF2α | human HIF-2α | GenBank:AAC51212 |
| coHIF2α | cow HIF-2α | GenBank:BAA78676 |
| dHIF2α | dog HIF-2α | GenBank:XM_531807 |
| cmHIF2α | chimpanzee HIF-2α | GenBank:XM_515449 |
| rHIF2α | rat HIF-2α | GenBank:CAB96612 |
| mHIF2α | mouse HIF-2α | GenBank:NP_034267 |
| xHIF1α | *Xenopus* HIF-1α | GenBank:CAB96628 |
| chHIF1α | chick HIF-1α | GenBank:BAA34234 |
| hHIF1α | human HIF-1α | GenBank:AAF20149 |
| coHIF1α | cow HIF-1α | GenBank:BAA78675 |
| mHIF1α | mouse HIF1-α | GenBank:NM_034561 |
| rHIF1α | rat HIF-1α | GenBank:CAA70701 |
| zHIF1α | zebrafish HIF-1α | GenBank:AAH46875 |
| gpHIF1α | scaleless carp HIF-1α | GenBank:AY745735 |
| gcHIF1α | grass carp HIF-1α | GenBank:AY450269 |
| rtHIF1α | rainbow trout HIF-1α | GenBank:AAK30364 |
| fuHIF1α | *Fugu* HIF-1α | Scaffold2916 |
| grHIF1α | grouper HIF-1α | GenBank:AY735010 |
| fARNT2 | *Fundulus* ARNT2 | GenBank:AAL95710 |
| zARNT2a | zebrafish ARNT2a | GenBank:AAG25919 |
| zARNT2b | zebrafish ARNT2b | GenBank:AAG25920 |
| zARNT2c | zebrafish ARNT2c | GenBank:AAG2592 |
| hARNT2 | human ARNT2 | GenBank:NP_055677 |
| mARNT2 | mouse ARNT2 | GenBank:NP_031514 |
| rARNT2 | rat ARNT2 | GenBank:NP_036913 |
| xARNT | *Xenopus* ARNT | GenBank:AAK68638 |
| chARNT | chick ARNT | GenBank:AAK25815 |
| hARNT1 | human ARNT1 | GenBank:NP_001659 |
| rARNT1 | rat ARNT1 | GenBank:NP_036912 |
| mARNT1 | mouse ARNT1 | GenBank:AAA56717 |
